# Nrf2 pathway mediates copper oxide nanoparticle-induced exacerbation of allergic asthma

**DOI:** 10.1016/j.redox.2026.104180

**Published:** 2026-04-20

**Authors:** Woong-Il Kim, Sin-Hyang Park, Ba-Reun Jin, So-Won Pak, Junhyeong Lee, Min-Jung Park, Changjong Moon, In-Sik Shin, Jong-Choon Kim

**Affiliations:** a Research Institute of Veterinary Medicine, Chonnam National University, Gwangju, 61186, Republic of Korea; b College of Veterinary Medicine and BK21 FOUR Program, Chonnam National University, Gwangju, 61186, Republic of Korea; c Center for Convergence Toxicology Research, Korea Institute of Toxicology, Daejeon, 34114, Republic of Korea

**Keywords:** Copper oxide nanoparticles, Asthma, Nuclear factor erythroid 2-related factor 2, Oxidative stress, Airway inflammation

## Abstract

Copper oxide nanoparticles (CuONPs), widely used across various industries, pose inhalation risks to industrial workers and consumers through respiratory exposure. Although exposure to CuONPs has been implicated in the initiation and progression of respiratory disorders, the molecular mechanisms underlying these effects remain poorly defined. In this study, we investigated the role of the nuclear factor erythroid 2–related factor 2 (Nrf2) signaling pathway in CuONP-induced respiratory toxicity and asthma exacerbation. Exposure to CuONPs resulted in pronounced inflammatory cell infiltration, elevated cytokine production, and excessive mucus secretion, accompanied by disrupted redox balance, as evidenced by increased malondialdehyde (MDA) levels and decreased glutathione (GSH) concentrations. These changes were associated with upregulation of Nrf2 and its downstream antioxidant enzymes, including heme oxygenase-1 (HO-1) and glutamate-cysteine ligase modifier subunit (GCLM). Although this antioxidant response is consistent with an expected oxidative stress–adaptive pathway, it was not sufficient to prevent CuONP-associated inflammatory and redox disturbances. Consistently, in NCI–H292 cells, CuONP treatment increased the expression of Nrf2, HO-1, and GCLM, whereas siRNA-mediated Nrf2 knockdown abrogated these inductions. In an ovalbumin (OVA)-induced asthma model, CuONP exposure further intensified airway inflammation and oxidative stress, despite elevated Nrf2 expression. However, adeno-associated virus (AAV)-mediated Nrf2 overexpression significantly attenuated CuONP-induced airway inflammatory responses and redox imbalance in asthmatic mice. Taken together, our results indicate that endogenous Nrf2 response is insufficient to counteract CuONP-driven asthma exacerbation, whereas pharmacological or genetic augmentation of Nrf2 signaling may constitute a viable strategy to alleviate nanoparticle-induced respiratory injury.

## Highlights

• CuONPs exacerbate allergic inflammation and oxidative stress in asthmatic mice.
• Endogenous Nrf2 activation is insufficient to counter CuONP toxicity.
• Nrf2 overexpression attenuates CuONP-driven inflammation and oxidative stress.
• Nrf2 functions as a key regulator of CuONP-induced asthma exacerbation.

## Graphical abstract

**Figure:**
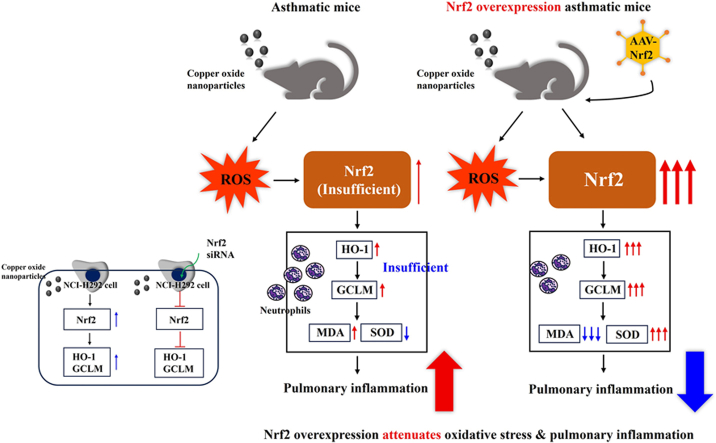

## 1 Introduction

Copper oxide nanoparticles (CuONPs) are extensively applied in diverse industrial sectors, including catalysis, electronics, and antimicrobial agents, owing to their unique physicochemical properties [[1], [2], [3]]. However, with the growing production and utilization of CuONPs, increasing concern has arisen regarding their potential health risks, particularly through inhalation, which represents the primary route of human exposure [4]. Due to their high surface area–to–volume ratio, CuONPs exhibit greater reactivity than bulk copper oxide and can induce marked oxidative stress and cytotoxicity [5,6]. While recent experimental studies have suggested that CuONPs may exacerbate respiratory diseases phenotypes in murine models [7,8], CuONP-specific clinical evidence in humans remains scarce. However, epidemiological studies indicated that transition metals, including copper, found in ambient particulate matter are associated with impaired pulmonary function and asthma exacerbation in humans [9,10]. Furthermore, occupational exposure to copper-containing metal fumes has been associated with respiratory symptoms; notably, these scenarios typically involve mixed-metal aerosols and are not specifically characterized as engineered CuONPs [11,12]. Collectively, these observations support the plausibility that inhaled copper-containing particulate exposures can contribute to respiratory morbidity and underscore the need to elucidate the mechanisms underlying CuONP-induced respiratory toxicity.

Asthma represents one of the most widespread chronic respiratory disorder globally, marked by clinical manifestations including coughing, airway hyperresponsiveness (AHR), wheezing, phlegm, and chest tightness, all of which contribute to substantial impairment in patients’ quality of life [13,14]. Its pathogenesis involves both genetic predisposition and environmental triggers, including air pollution and chemical exposure [15,16]. Upon allergen exposure, type 2 cytokines such as interleukin (IL)-4, IL-5, and IL-13 are secreted, driving Th2-dominant immune responses and promoting immunoglobulin (Ig)E production, which in turn induces eosinophilic inflammation [17,18]. This cascade contributes to hallmark features of asthma, including eosinophilia, AHR, and mucus hypersecretion [19]. In addition, eosinophil infiltration and the resulting overproduction of reactive oxygen species (ROS) disrupt antioxidant defenses, inducing oxidative stress that further aggravates airway inflammation and hyperresponsiveness [20]. As oxidative stress is a critical determinant in asthma development and progression, environmental exposures that induce ROS are increasingly recognized as important contributing factors. Among them, airborne nanoparticles have emerged as major culprits, especially in the context of rising air pollution. Recent studies have shown that such exposures can initiate or worsen asthma symptoms [[21], [22], [23]]. However, the mechanisms by which nanoparticles influence asthma development and exacerbation remain largely unclear.

Nuclear factor erythroid 2–related factor 2 (Nrf2) is a master transcriptional regulator that coordinates cellular defenses against oxidative stress by inducing the expression of a broad array of cytoprotective genes [24,25]. Upon activation, Nrf2 translocates to the nucleus and promotes transcription of antioxidant genes such as heme oxygenase-1 (HO-1) and NAD(P)H quinone oxidoreductase 1 (NQO1), which encode enzymes that detoxify reactive intermediates and reduce oxidative damage [26]. Nrf2 also regulates genes involved in glutathione (GSH) synthesis and regeneration, including glutamate-cysteine ligase modifier subunit (GCLM) and glutathione reductase (GR), thereby enhancing cellular antioxidant capacity [27]. Nrf2 has been implicated in asthma pathogenesis through its regulatory roles in oxidative stress and inflammation [28,29]. Clinically, patients with severe asthma often exhibit defective antioxidant defenses, including dysregulated Nrf2 signaling, which correlates with disease severity and chronic oxidative stress [30]. However, its contribution to asthma exacerbation induced by nanoparticle-driven oxidative stress remains controversial. While CuONP exposure has been associated with activation of the Nrf2 pathway [31,32], it remains unclear whether CuONP-induced Nrf2 activation is sufficient to counteract the excessive oxidative stress observed in exacerbated asthma. Thus, the key unresolved question is not whether CuONPs activate oxidative stress–responsive Nrf2 signaling, but whether this endogenous response is functionally sufficient to protect the asthmatic airway from nanoparticle-aggravated injury. This ambiguity underscores the need for further investigation into the precise role of Nrf2 in CuONP-induced asthma exacerbation.

Therefore, the present study aimed to elucidate the role of Nrf2 in CuONP-induced asthma exacerbation by evaluating airway inflammation, oxidative stress, and histopathological changes. To elucidate this mechanism, we used both an acute CuONP exposure model and an ovalbumin (OVA)-induced asthma model, and further employed Nrf2-overexpressing mice and Nrf2-knockdown NCI–H292 cells.

## 2 Material & methods

### 2.1 Nanoparticles

CuONPs were obtained from Sigma-Aldrich (St. Louis, MO, USA). CuONPs were suspended in phosphate-buffered saline (PBS) and sonicated for 5 min using an ultrasonic bath sonicator to facilitate dispersion (Sungdong, Seoul, Republic of Korea; 40 kHz, 150 W).

The morphology and particle size of CuONPs were analyzed using transmission electron microscopy (TEM; JEM-2100F, JEOL, Tokyo, Japan) and scanning electron microscopy (SEM; Zeiss Gemini 500, Carl Zeiss, Jena, Germany). To characterize the surface charge of CuONPs, the zeta potential was measured using a zeta potential analyzer (ELSZeno, Otsuka Electronics, Tokyo, Japan). For zeta potential and hydrodynamic size distribution measurements, CuONPs were dispersed in distilled water (1 mg/mL) and sonicated for 30 min prior to analysis. Hydrodynamic size distribution was evaluated by dynamic light scattering (DLS) using the same instrument. Elemental composition was assessed by energy-dispersive X-ray spectroscopy (EDS) coupled with TEM. Endotoxin contamination in CuONP suspensions (0.25–2 μg/mL) was evaluated using a recombinant Factor C (rFC) assay kit (AcroBiosystems, Newark, DE, USA).

### 2.2 Animals and experimental design

Specific pathogen-free (SPF) female BALB/c mice (6 weeks) were purchased from Samtako Co. (Osan, Republic of Korea) and acclimatized for one week prior to experimentation. All animals were maintained under SPF conditions. Experimental protocols were approved by the Institutional Animal Care and Use Committee (IACUC) of Chonnam National University (CNU IACUC-YB-2021-32 and CNU IACUC-YB-2022-74) and conducted in compliance with the NIH Guide for the Care and Use of Laboratory Animals.

To evaluate the respiratory toxicity of CuONPs, mice were randomly assigned to two groups (n = 6 per group): normal control (NC) and CuONP-treated (0.5 mg/kg). On days 1, 3, and 5, mice were lightly anesthetized with isoflurane (Isotory®, Troikaa Pharmaceuticals Ltd., Gujarat, India) and intranasally administered either PBS (50 μL/mouse) or CuONPs (0.5 mg/kg in 50 μL of PBS). The dose of CuONPs (0.5 mg/kg) was selected based on previous studies [8,33] and was used as a biologically active exposure level to evaluate mechanistic effects in the asthma exacerbation model. The administered dose was calculated according to each mouse's body weight measured immediately before administration (Fig. S1A).

To assess the effects of CuONPs on asthma exacerbation, mice were divided into three groups (n = 6 per group): NC, OVA, and OVA + CuONPs. Sensitization was performed on days 1 and 15 via intraperitoneal injection of OVA (20 μg/mouse; Sigma-Aldrich) and aluminum hydroxide (2 mg/mouse; Sigma-Aldrich) in 200 μL PBS. Asthma was induced by exposure to 1% OVA aerosol for 1 h on days 21, 23, and 25 using an ultrasonic nebulizer (NE-U12, Omron, Kyoto, Japan) according to a previously described protocol [34,35]. CuONPs were administered intranasally on days 20, 22, and 24. On day 26, following tracheotomy, AHR was assessed the FlexiVent system (SCIREQ, Montreal, Canada) by measuring total respiratory system resistance (Rrs) in response to aerosolized PBS and increasing concentrations of methacholine (10, 20, and 40 mg/mL) (Fig. S1B).

To determine the role of Nrf2 in CuONP-induced asthma exacerbation, an Nrf2 overexpression model was established using adeno-associated virus (AAV) serotype 2/8, based on a previous study [36]. Seven days before the initiation of the OVA protocol, mice were intranasally administered AAV encoding either green fluorescent protein (GFP) or Nrf2. Mice were then divided into NC, OVA, and OVA + CuONPs groups (n = 6 per group). The subsequent procedures for asthma induction, CuONP administration, and AHR measurement were conducted as described above (Fig. S1C).

### 2.3 DNA cloning, production, and purification of AAV

To enable Nrf2 overexpression via adeno-associated virus (AAV), the mouse Nfe2l2 gene (NM_010902.4), tagged with FLAG and driven by the CMV promoter, was synthesized and cloned into an AAV expression vector by VectorBuilder (Chicago, IL, USA). The pAAV vector carrying the target gene, the packaging plasmid pAAV2/8, and the helper plasmid pAdDeltaF6 (Addgene, Watertown, MA, USA) were co-transfected into HEK293 cells at a 1:1:1 M ratio. Three days post-transfection, the cells were harvested and lysed in AAV lysis buffer (150 mM NaCl, 20 mM Tris, pH 8.0). Benzonase (Sigma-Aldrich) was added to the lysates to degrade nucleic acids, followed by centrifugation to remove cellular debris. The viral particles in the supernatant were purified using iodixanol gradient ultracentrifugation (15%, 25%, 40%, and 60%) in QuickSeal tubes (Hitachi, Tokyo, Japan). The samples were centrifuged at 280,000×*g* for 3 h at 14 °C using a Himac CP80WX ultracentrifuge equipped with a P90AT rotor (Hitachi). The viral fraction at the 40%–60% interface was collected, concentrated using Amicon Ultra-15 centrifugal filter units (Sigma-Aldrich), and washed three times with PBS. Finally, mice were intratracheally administered either purified AAV-Nrf2 or control AAV-GFP at a dose of 2 × 10^11^ viral particles per mouse.

### 2.4 Bronchoalveolar lavage fluid and serum analysis

Two days after the final administration of CuONPs or OVA, whole blood was collected from the inferior vena cava under anesthesia, and the mice were euthanized. Tracheotomy was performed, and the left bronchus was ligated to preserve the left lung for histological analysis. Bronchoalveolar lavage fluid (BALF) was collected by instilling 700 μl of sterile PBS into right lung via a cannula and withdrawing it twice. BALF was centrifuged at 300×*g* for 10 min at 4 °C, and the supernatant was stored for cytokine analysis using commercially available enzyme-linked immunosorbent assay (ELISA) kits to quantify IL-1β, IL-6, tumor necrosis factor (TNF)-α, IL-4, IL-5, and IL-13 (R&D Systems, Minneapolis, MN, USA; Cat. No. MLB00C, M6000B, MTA00B, M4000B, M5000, and M1300CB, respectively). The cell pellet was resuspended in 600 μL PBS, and total cell counts were obtained with an automated cell counter (Cell Countess III, Thermo Fisher Scientific, Waltham, MA, USA). For differential cell counts, BALF cells were cytocentrifuged onto glass slides (Hanil Science Industrial Co., Ltd., Seoul, Republic of Korea) and stained with Diff-Quik (Thermo Fisher Scientific). Inflammatory cell types were identified and counted under a light microscope at 200× magnification. Differential cells were calculated based on the total cell counts obtained with the Cell Countess III. To measure total serum IgE and OVA-specific IgE levels, blood collected from the inferior vena cava were centrifuged at 1800×*g* for 10 min at 4 °C, and IgE concentrations were quantified using a commercial ELISA kit (BioLegend, San Diego, CA, USA; Cat. No. 432404).

### 2.5 Oxidative stress analysis

Lung tissue oxidative stress markers were assessed by quantifying malondialdehyde (MDA) levels, GSH content, and superoxide dismutase (SOD) activity using commercially available kits (Cayman Chemical, Ann Arbor, MI, USA; Cat. No. 700870, 703002, 706002, respectively).

### 2.6 Histopathology and immunohistochemistry

The left lung lobe, which had been ligated prior to BALF collection, was fixed in 10% neutral buffered formalin for three days. The tissue was then processed, embedded in paraffin, and sectioned at 4 μm thickness. Sections were stained with hematoxylin and eosin (H&E; Sigma-Aldrich) to assess airway inflammation, and with periodic acid–Schiff (PAS; IMEB Inc., San Marcos, CA, USA) solution to evaluate mucus production. For quantitative analysis of inflammation, the total inflamed area was measured under 100 × magnification. Mucus production was assessed by quantifying the PAS-positive area within the bronchial epithelium. To evaluate protein expression in lung tissue, immunohistochemistry (IHC) was performed using a commercial avidin–biotin complex kit (Vector Laboratories, Burlingame, CA, USA; Cat. No. PK-6100). The primary antibodies used were anti-Nrf2 (1:200 dilution; Thermo Fisher Scientific; Cat. No. PA5-27882) and anti–HO–1 (1:200 dilution; Abcam, Cambridge, UK; Cat. No. ab13243). Quantitative histological analyses were performed using ImageJ software version 1.51. The inflammation index was calculated as the percentage of the area occupied by inflammatory cell infiltration relative to the total analyzed tissue area. The mucus production index was calculated as the percentage of PAS-positive stained area relative to the total analyzed area. Immunoreactivity for Nrf2 and HO-1 was quantified as the percentage of DAB-positive area relative to the total analyzed area. Histological image quantification was performed by an investigator blinded to group allocation.

### 2.7 Western blotting

Homogenized lung tissue lysates (20 μg per sample) were separated by 8% or 12% SDS-polyacrylamide gel electrophoresis and transferred to polyvinylidene fluoride membranes (Millipore, Burlington, MA, USA). After blocking with 5% skim milk at room temperature for 3 h, the membranes were incubated with primary antibodies (1:1000 dilution) at 4 °C for 12 h, followed by incubation with HRP-conjugated secondary antibodies (1:10000 dilution) at room temperature for 2 h. Protein bands were visualized using an enhanced chemiluminescence kit (Thermo Fisher Scientific) and imaged using the Chemi-Doc system (Bio-Rad Laboratories, Hercules, CA, USA). Primary antibodies against Nrf2 (Thermo Fisher Scientific; Cat. No. PA5-27882), HO-1 (Abcam; Cat. No. ab13243), GCLM (Abcam; Cat. No. ab126704), and β-actin (Cell Signaling Technology, Danvers, MA, USA; Cat. No. 4967S) were used. Densitometric analysis was performed using ImageJ software version 1.51.

### 2.8 Cell culture and cell viability assay

The NCI–H292 human airway epithelial cell line was obtained from the American Type Culture Collection (ATCC, Manassas, VA, USA). Cells were cultured in RPMI-1640 medium (WELGENE, Gyeongsan, Republic of Korea) supplemented with 10% fetal bovine serum (FBS) and 1% penicillin–streptomycin at 37 °C in a humidified atmosphere containing 5% CO_2_. For the cell viability assay, cells were seeded in 96-well plates at a density of 1 × 10^4^ cells/well and allowed to attach for 24 h. Cells were then treated with various concentrations of CuONPs for 24 h. Subsequently, 10 μL of EZ-Cytox solution (DoGenBio, Seoul, Republic of Korea; Cat. No. EZ-500) was added to each well, and plates were incubated for 4 h. Absorbance was measured at 450 nm using a microplate reader (TECAN, Männedorf, Switzerland).

### 2.9 Intracellular ROS detection in NCI–H292 cells

Intracellular ROS levels were measured using the DCFDA/H_2_DCFDA cellular ROS assay kit (Abcam; Cat. No. ab113851) according to the manufacturer's instructions. Cells were seeded in black, clear-bottom 96-well plates at a density of 2.5 × 10^3^ cells/well and incubated for 24 h. Cells were then incubated with 100 μL of 20 μM DCFDA solution at 37 °C for 45 min in the dark. After washing with buffer to remove excess dye, cells were treated with various concentrations of CuONPs. Fluorescence intensity was measured using a microplate reader (TECAN) at 485 nm (excitation) and 535 nm (emission).

### 2.10 Immunofluorescence

Cells were seeded onto coverslips and allowed to adhere overnight. The next day, cells were treated with either CuONPs (2 μg/mL) or fresh medium (control) for 12 h, followed by immunofluorescence staining. After treatment, cells were fixed and incubated with the primary antibody anti-Nrf2 (1:200 dilution; Thermo Fisher Scientific; Cat. No. PA5-27882) at 4 °C overnights, and then with the secondary antibody, anti-rabbit IgG-FITC (1:100, Sigma-Aldrich; Cat. No. F0382), at room temperature for 2 h. Nuclei were counterstained with DAPI (Thermo Fisher Scientific; Cat. No. P36931), and fluorescence images were acquired using a confocal microscope (ZEISS, Oberkochen, Germany) at 630 × magnification.

### 2.11 Small interfering RNA transfection

Cells were seeded at a density of 4 × 10^4^ cells/dish in 60 × 15 mm cell culture dishes and cultured for 24 h Nrf2-specific siRNA or scrambled control siRNA was transfected using Lipofectamine™ RNAiMAX reagent (Thermo Fisher Scientific; Cat. No. 13778100), according to the manufacturer's instructions. After knockdown of Nrf2 expression, cells were treated with CuONPs (2 μg/mL) or fresh medium (control) and harvested after 6 h.

### 2.12 Statistical analysis

Data are presented as mean ± standard deviation (SD). Normality was assessed using the Shapiro–Wilk test in GraphPad Prism (GraphPad Software, San Diego, CA, USA). For comparisons between two groups, an unpaired two-tailed Student's t-test was used. For single-factor comparisons among more than two groups, one-way analysis of variance (ANOVA) followed by Tukey's post hoc test was applied. For factorial designs, two-way ANOVA with appropriate multiple-comparison testing was used. AHR to methacholine challenge was analyzed using two-way repeated-measures ANOVA (group × methacholine concentration). A *p*-value <0.05 was considered statistically significant.

## 3 Results

### 3.1 Physicochemical characterization of CuONPs

**Fig. 1:**
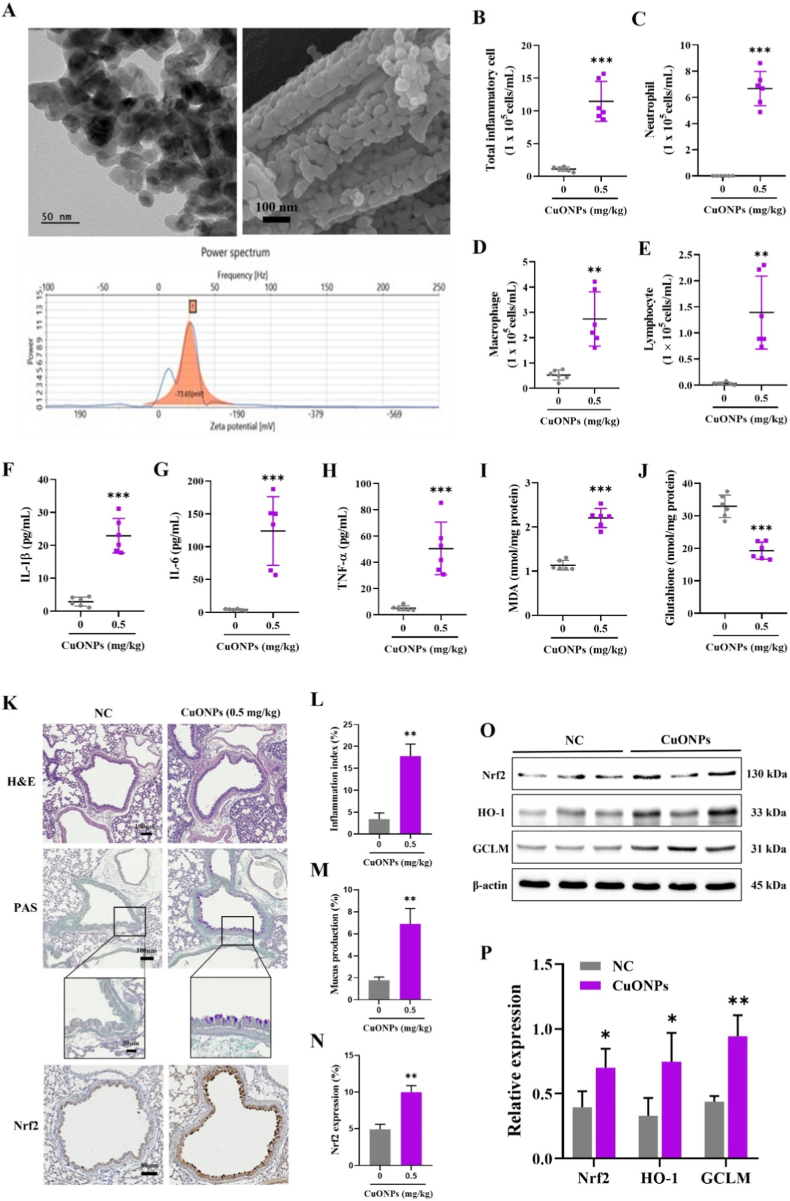
Physicochemical characterization of CuONPs and pathophysiological changes in CuONP-exposed mice. (A) CuONP morphology assessed by TEM and SEM, and zeta potential measured using zeta potential analyzer. Scale bar = 50 nm (TEM), 100 nm (SEM). (B–E) Total and differential inflammatory cell counts in BALF. (F–H) IL-1β, IL-6, and TNF-α levels in BALF, measured by ELISA. (I and J) MDA and GSH levels in lung tissue. (K) Representative lung sections stained with H&E (scale bar = 100 μm), PAS for mucus production (scale bars = 100, 30 μm), and Nrf2 IHC (scale bar = 80 μm). (L) Inflammation index. (M) Mucus production index. (N) Quantification of Nrf2-positive area. (O and P) Protein expression of Nrf2, HO-1, and GCLM analyzed by Western blotting and densitometric analysis. Data are presented as means ± SD (n = 6 mice/group for panels B–J; n = 3 mice/group for panels K–P). ∗*p*< 0.05, ∗∗*p*< 0.01, and ∗∗∗*p* < 0.001 versus NC group.

SEM and TEM analyses showed that CuONPs were predominantly spherical, with a primary particle diameter of 45.22 ± 10.44 nm (Fig. 1A). The zeta potential of CuONPs measured in distilled water was −73.65 mV (Fig. 1A), indicating a strongly negative surface charge and suggesting substantial electrostatic repulsion under the measurement conditions. In addition, DLS analysis revealed a highly polydisperse hydrodynamic size distribution with micron-scale agglomerated populations in water (Fig. S2A), indicating that dispersion-state behavior may vary depending on the medium and handling conditions. EDS confirmed copper and oxygen as the predominant elements in the CuONP samples (Fig. S2B). Endotoxin contamination assessed by the rFC assay was below the limit of detection across the tested CuONP concentrations (Fig. S3).

### 3.2 Effects of CuONPs exposure on mice

Mice exposed to CuONPs exhibited a significant increase in total inflammatory cells, including neutrophils, macrophages, and lymphocytes, in BALF compared with the NC group (Fig. 1B–E). Pro-inflammatory cytokine levels, including IL-1β, IL-6, and TNF-α, were also markedly elevated following CuONP treatment (Fig. 1F–H). CuONP exposure also significantly increased MDA levels, whereas GSH levels were markedly reduced in lung tissue (Fig. 1I and J). Histopathological examination demonstrated pronounced inflammatory cell infiltration, mucus hypersecretion, and upregulated Nrf2 expression in the bronchial epithelium and alveolar regions of CuONP-exposed mice (Fig. 1K–N). Furthermore, Western blot analysis demonstrated a significant upregulation of Nrf2 and antioxidant-related proteins, including HO-1 and GCLM, in lung tissue following CuONP exposure (Fig. 1O and P).

### 3.3 Effects of CuONP exposure on the exacerbation of allergic airway inflammation in OVA-induced asthmatic mice

**Fig. 2:**
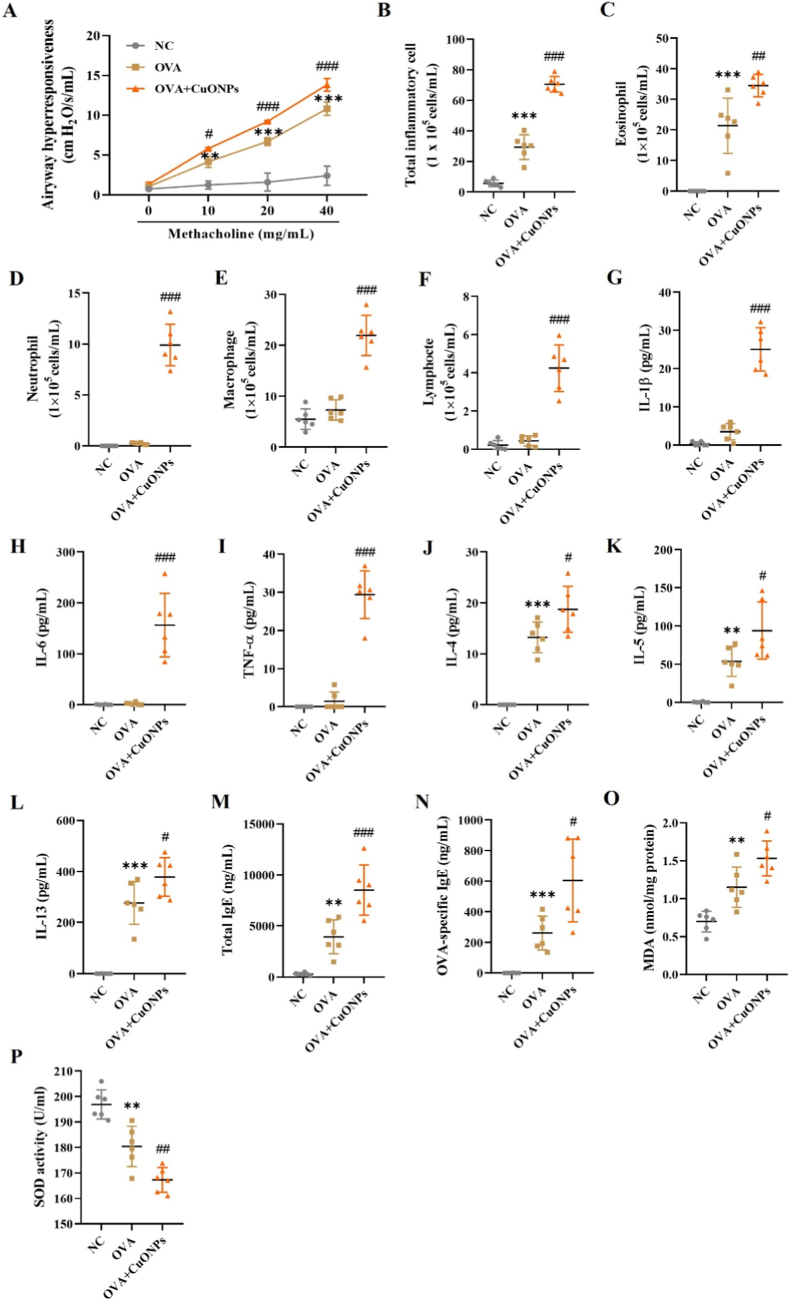
Effects of CuONPs on allergic inflammation and oxidative stress in OVA-induced asthmatic mice. (A) Airway hyperresponsiveness assessed as total respiratory system resistance in response to methacholine challenge (10, 20, and 40 mg/mL). (B–F) Total and differential inflammatory cell counts in BALF. (G–L) IL-1β, IL-6, TNF-α, IL-4, IL-5, and IL-13 levels in BALF, measured by ELISA. (M and N) Total IgE and OVA specific IgE levels in serum. (O and P) MDA levels and SOD activity in lung tissue. Data are presented as means ± SD (n = 6 mice/group). ∗∗*p*< 0.01 and ∗∗∗*p* < 0.001 versus NC group. ^#^*p* < 0.05, ^##^*p* < 0.01, and ^###^*p* < 0.001 versus OVA group.

Mice in the OVA group exhibited significantly increased Rrs at methacholine concentrations of 10 mg/mL or higher compared to the NC group, and this response was further amplified by CuONP exposure (Fig. 2A). The OVA group also showed a significant increase in total inflammatory cells, particularly eosinophils, in BALF compared with the NC group. CuONP exposure in asthmatic mice further elevated the numbers of eosinophils, neutrophils, macrophages, and lymphocytes compared with the OVA group (Fig. 2B–F). Pro-inflammatory cytokines (IL-1β, IL-6, TNF-α) and type 2 cytokines (IL-4, IL-5, IL-13) were significantly elevated in BALF from CuONP-exposed asthmatic mice relative to the OVA group (Fig. 2G–L). Moreover, serum total IgE and OVA specific IgE concentrations were markedly increased following CuONP exposure (Fig. 2M and N). Lung tissue analysis revealed that MDA levels, an indicator of lipid peroxidation, were significantly increased in both the OVA and CuONP-exposed groups, with more pronounced elevation in the latter. In contrast, SOD activity was significantly decreased in CuONP-exposed group (Fig. 2O and P).

### 3.4 Effects of CuONP exposure on the histopathological alterations and Nrf2-associated protein in OVA-induced asthmatic mice

**Fig. 3:**
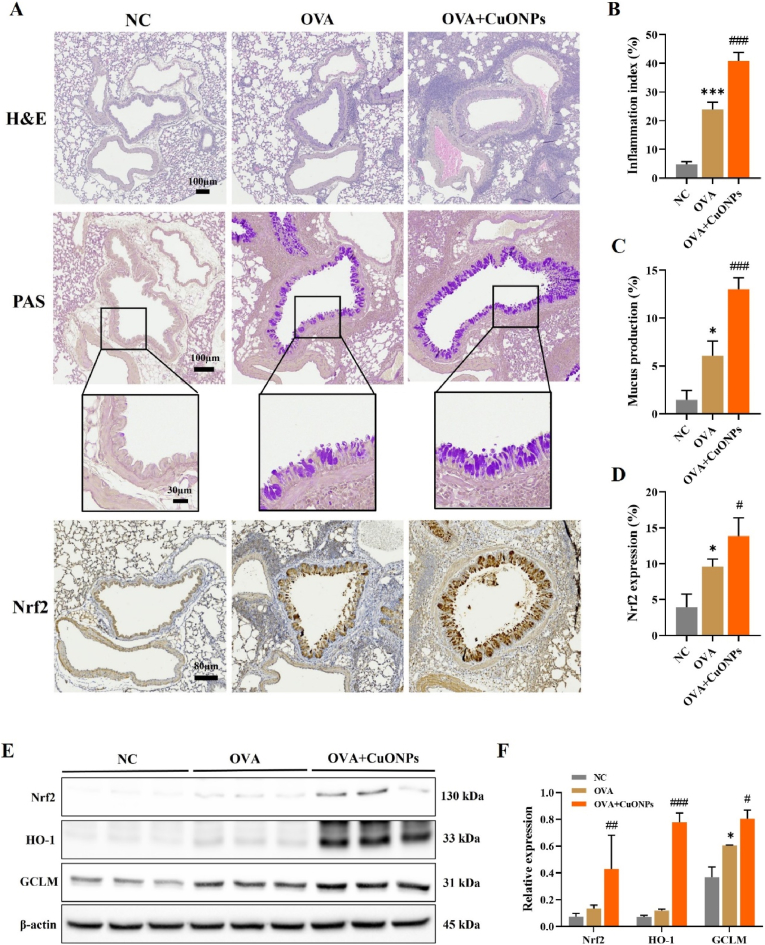
Effects of CuONPs on inflammatory cell infiltration, mucus production, and Nrf2-related antioxidant proteins in OVA-induced asthmatic mice. (A) Representative lung sections stained with H&E (scale bar = 100 μm), PAS for mucus production (scale bars = 100, 30 μm), and Nrf2 IHC (scale bar = 80 μm). (B) Inflammation index. (C) Mucus production index. (D) Quantification of Nrf2-positive area. (E and F) Protein expression of Nrf2, HO-1, and GCLM analyzed by Western blotting and densitometric analysis. Data are presented as means ± SD (n = 3 mice/group). ∗*p*< 0.05 and ∗∗∗*p* < 0.001 versus NC group. ^#^*p* < 0.05, ^##^*p* < 0.01, and ^###^*p* < 0.001 versus OVA group.

Histopathological evaluation demonstrated that OVA exposure alone induced pronounced inflammatory cell infiltration, mucus hypersecretion, and enhanced Nrf2 expression in the bronchial epithelium and alveolar regions compared with the NC group. These pathological changes were further exacerbated in the CuONP-exposed group (Fig. 3A–D). In parallel, Western blot analyses revealed that CuONP exposure significantly upregulated Nrf2 and its downstream antioxidant proteins, including HO-1 and GCLM, in lung tissue compared with the OVA group (Fig. 3E and F).

### 3.5 Effects of Nrf2 overexpression on CuONP-induced exacerbation of allergic airway inflammation in asthmatic mice

**Fig. 4:**
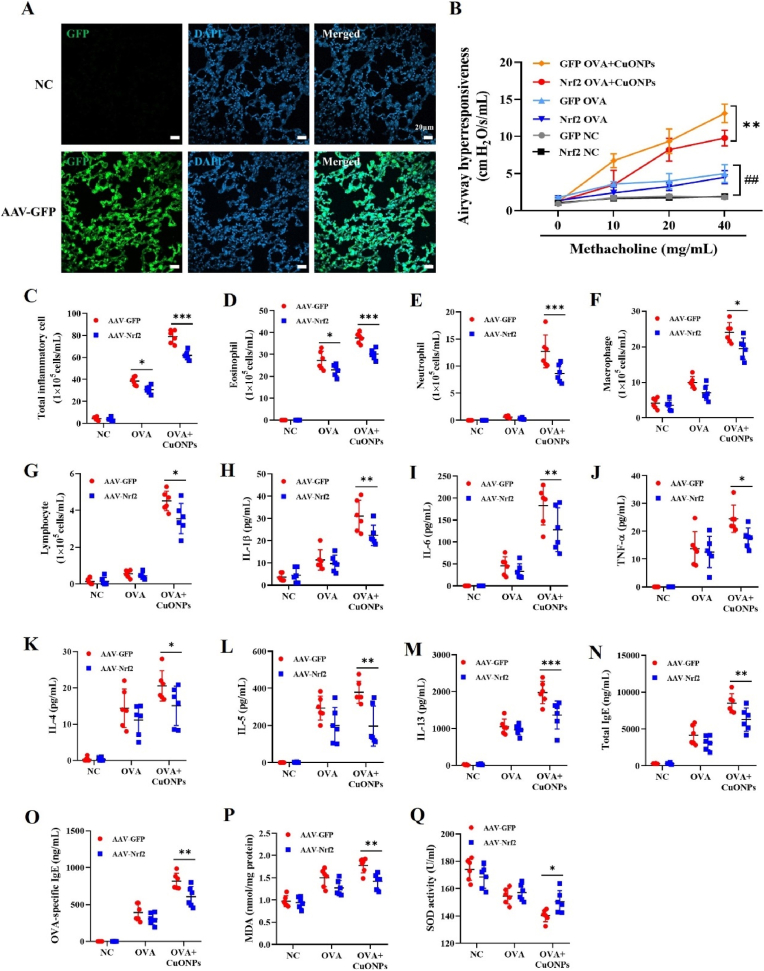
Effects of Nrf2 overexpression on allergic inflammation and oxidative stress in CuONP-exposed asthmatic mice. (A) Immunofluorescence analysis of lung tissue from mice administered PBS or AAV2/8-GFP via intratracheal instillation. (B) Airway hyperresponsiveness assessed as total respiratory system resistance in response to methacholine challenge (10, 20, and 40 mg/mL). (C–G) Total and differential inflammatory cell counts in BALF. (H–M) IL-1β, IL-6, TNF-α, IL-4, IL-5, and IL-13 levels in BALF, measured by ELISA. (N and O) Total IgE and OVA specific IgE levels in serum. (P and Q) MDA levels and SOD activity in lung tissue. Data are presented as means ± SD (n = 3 mice/group for panels A; n = 6 mice/group for panels B–Q). In panel B, ^##^*p*< 0.01 indicates significant differences between GFP-NC and GFP-OVA, and ∗∗*p*< 0.01 indicates significant differences between GFP-OVA + CuONPs and Nrf2-OVA + CuONPs. For panels C–Q, ∗*p*< 0.05, ∗∗*p*< 0.01, and ∗∗∗*p* < 0.001 indicate significant differences between AAV-GFP and AAV-Nrf2 within each condition.

We confirmed successful Nrf2 overexpression in the lungs using an AAV system, as visualized by GFP expression (Fig. 4A). Nrf2-overexpressing mice in the OVA + CuONPs group exhibited significantly reduced Rrs in response to high-dose methacholine compared with control mice overexpressing GFP (Fig. 4B). In asthmatic mice exposed to CuONPs, Nrf2 overexpression significantly reduced inflammatory cells, including eosinophils, neutrophils, macrophages, and lymphocytes, in BALF (Fig. 4C–G). This was accompanied by a marked reduction in both pro-inflammatory cytokines (IL-1β, IL-6, TNF-α) and type 2 cytokines (IL-4, IL-5, IL-13), as well as serum total IgE and OVA specific IgE levels (Fig. 4H–O). Furthermore, Nrf2 overexpression markedly suppressed MDA levels, a marker of oxidative stress, and significantly restored SOD activity in the lungs of CuONP-exposed asthmatic mice (Fig. 4P and Q).

### 3.6 Effects of Nrf2 overexpression on CuONP-induced histopathological alterations and antioxidant protein expression in asthmatic mice

**Fig. 5:**
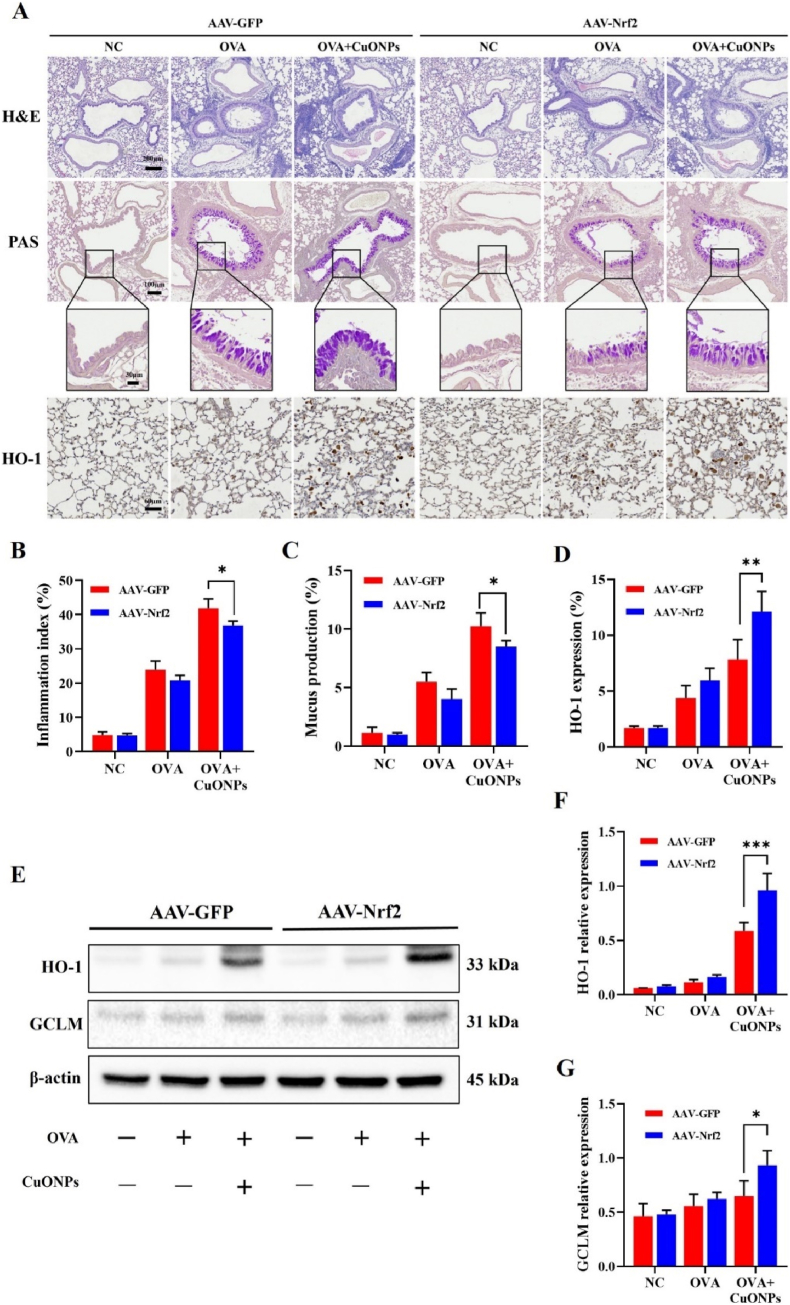
Effects of Nrf2 overexpression on inflammatory cell infiltration, mucus production, and HO-1 expression in CuONP-exposed asthmatic mice. (A) Representative lung sections stained with H&E (scale bar = 100 μm), PAS for mucus production (scale bar = 100, 30 μm), and HO-1 IHC (scale bar = 80 μm). (B) Inflammation index. (C) Mucus production index. (D) Quantification of HO-1-positive area. (E–G) Protein expression of HO-1 and GCLM analyzed by Western blotting and densitometric analysis. Data are presented as means ± SD (n = 3 mice/group). ∗*p*< 0.05, ∗∗*p*< 0.01, and ∗∗∗*p* < 0.001 indicate significant differences between AAV-GFP and AAV-Nrf2 within each condition.

Nrf2 overexpression attenuated CuONP-induced histopathological changes in OVA-induced asthmatic mice, including inflammatory cell infiltration, mucus hypersecretion, and HO-1 expression in the bronchial epithelium and alveolar regions, compared with the AAV-GFP control group (Fig. 5A–D). Consistently, Western blot analysis demonstrated that Nrf2 overexpression significantly increased the expression of antioxidant-related proteins, including HO-1 and GCLM, in the lungs of CuONP-exposed asthmatic mice (Fig. 5E–G).

### 3.7 Effects of CuONPs on cellular responses and signaling pathways in NCI–H292 cells

**Fig. 6:**
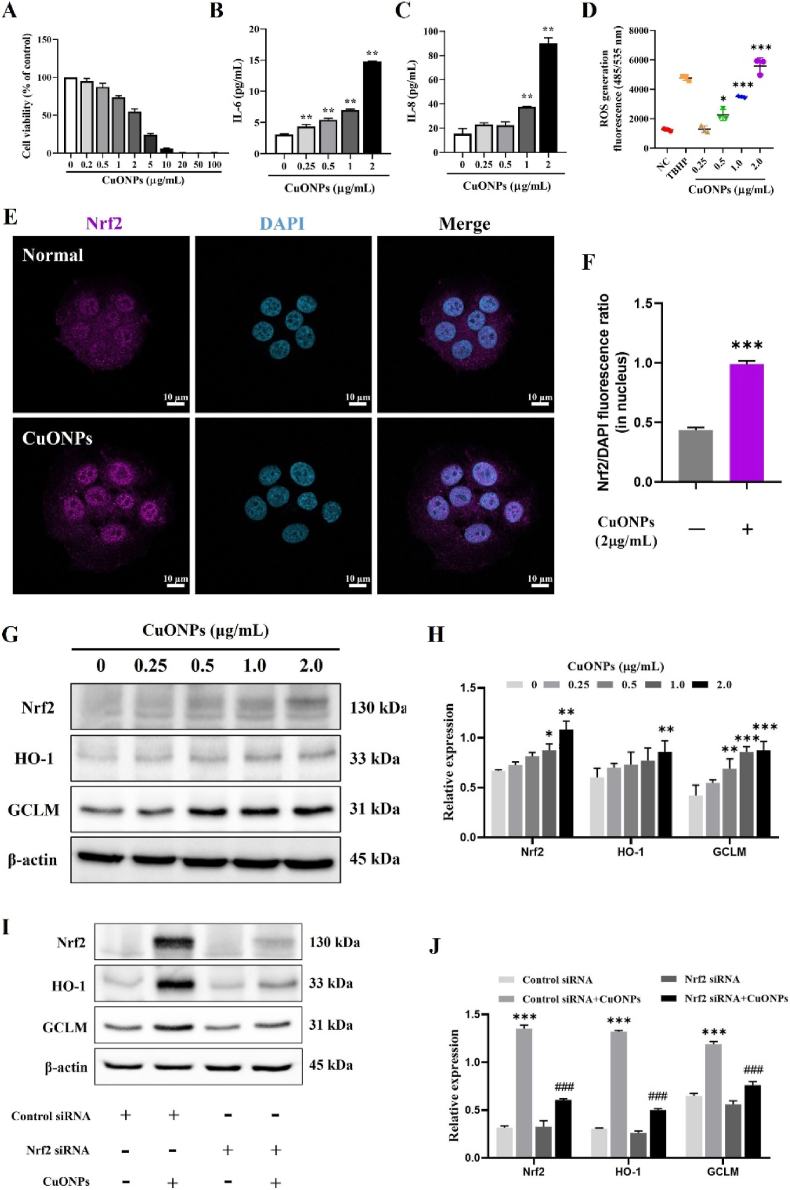
Effects of CuONPs on inflammatory response, oxidative stress and Nrf2 pathway activation in NCI–H292 cells. (A) Cell viability was assessed after 24 h exposure to CuONPs. (B and C) IL-6 and IL-8 levels were measured by ELISA. (D) Intracellular ROS generation was detected using DCFDA assay. (E and F) Nrf2 expression was visualized via immunofluorescence and quantified based on nuclear fluorescence intensity ratio. Scale bar = 10 μm. (G and H) Expression levels of Nrf2, HO-1 and GCLM were analyzed by Western blotting and densitometric analysis. (I and J) Protein expression of Nrf2, HO-1, and GCLM analyzed by Western blotting and densitometric analysis in cells with or without Nrf2 knockdown using siRNA. Data are presented as means ± SD (n = 3). ∗*p*< 0.05, ∗∗*p*< 0.01, and ∗∗∗*p* < 0.001 versus NC group. ^#^*p* < 0.05, ^##^*p* < 0.01, and ^###^*p*< 0.001 versus siRNA + CuONPs group.

As shown in Figs. 6A and 24 h exposure to CuONPs reduced the viability of NCI–H292 cells in a concentration-dependent manner. Cell viability was relatively maintained at concentrations up to 2.0 μg/mL, whereas a marked decrease was observed at ≥5.0 μg/mL, indicating substantial cytotoxicity at higher concentrations. Therefore, 2.0 μg/mL was selected as the maximum concentration for subsequent in vitro experiments to minimize confounding effects attributable to extensive cell death. CuONP-treated cells exhibited a significant, concentration-dependent increase in IL-6 and IL-8 levels compared with untreated control cells (Fig. 6B and C). In addition, treatment with CuONPs markedly elevated ROS production (Fig. 6D) and significantly promoted the nuclear translocation of Nrf2 (Fig. 6E and F). Furthermore, CuONP exposure resulted in a significant upregulation of Nrf2, HO-1, and GCLM protein expression compared with the control group (Fig. 6G and H). However, siRNA-mediated knockdown of Nrf2 effectively abolished the CuONP-induced upregulation of these proteins (Fig. 6I and J).

## 4 Discussion

The increasing application of nanomaterials across diverse industrial fields has heightened concerns regarding their possible adverse effects on human health risks [37,38]. Among these, CuONPs have garnered widespread industrial application owing to their distinctive physicochemical properties, prompting growing awareness regarding their health risks [[1], [2], [3]]. Inhalation represents the principal route of CuONP exposure, and their nanoscale dimensions enable deep pulmonary penetration and potential translocation into the bloodstream, potentially triggering immune responses and systemic toxicity [4,39,40]. Compared with non-nanoscale copper oxide, CuONPs may exhibit greater biological reactivity because their small size and high surface area can enhance cellular interaction, redox cycling, and copper ion dissolution [5,6]. Although the present study did not directly compare CuONPs with bulk CuO, these nanoparticle-specific properties provide a plausible basis for the pronounced oxidative and inflammatory responses observed in sensitized airways. CuONPs have been shown to induce respiratory inflammation through excessive ROS production, and several studies suggest they may exacerbate preexisting respiratory diseases such as asthma [23,[41], [42], [43]]. However, despite these findings, the molecular mechanisms by which CuONPs exacerbate asthma remain poorly defined. Here, we systematically characterized the physicochemical properties of CuONPs, assessed their pulmonary toxicity, and examined their effects on the Nrf2 pathway in an asthma model. Our findings showed that CuONP exposure increased Nrf2 expression and its downstream effectors HO-1 and GCLM in asthmatic mice but did not alleviate airway inflammation, mucus secretion, or oxidative stress. AAV-mediated overexpression of Nrf2, however, markedly attenuated these pathological changes, and this was further supported by Nrf2 knockdown results in NCI–H292 cells.

CuONPs have been shown to exert cytotoxic and pro-inflammatory effects primarily by disrupting intracellular redox balance [44,45]. Following cellular uptake, CuONPs can accumulate in lysosomes and mitochondria, where they release copper ions that catalyze redox cycling reactions. This leads to the formation of highly reactive free radicals and oxidative species that inflict damage on cellular components, including membranes, proteins, and DNA [46,47]. In the respiratory system, these effects compromise epithelial integrity, promote the release of inflammatory cytokines, and facilitates immune cell recruitment, thereby exacerbating airway inflammation and tissue injury. Given these oxidative mechanisms of CuONP toxicity, the cellular antioxidant defense machinery, with the Nrf2 signaling axis as a central component, is essential for preserving redox equilibrium and mitigating oxidative stress. Upon activation by oxidative stress, Nrf2 dissociates from its cytoplasmic repressor KEAP1 and translocates to the nucleus, where it induces expression of cytoprotective genes such as HO-1, GCLM, and NQO1, thereby enhancing cellular resilience to redox imbalance [48]. In line with this, our study demonstrated that CuONP exposure upregulated Nrf2 and its downstream antioxidant enzymes in asthmatic mice. However, despite this endogenous activation, CuONP-exposed mice exhibited persistent eosinophilic inflammation, elevated cytokine production, and enhanced mucus secretion, suggesting that the intrinsic activation of Nrf2 may be insufficient to counteract the magnitude of oxidative and inflammatory stress induced by CuONPs in a disease-sensitized airway milieu. In this respect, the observation that CuONPs activate oxidative stress–responsive Nrf2 signaling is confirmatory and consistent with prior literature on metal nanoparticle toxicity [31,32]. The incremental advance of the present study lies in showing that this endogenous antioxidant response is functionally insufficient in the asthmatic airway and that exogenous enhancement of Nrf2 signaling confers protection against CuONP-exacerbated pathology. Thus, the mechanistic significance of the present study lies not in documenting Nrf2 induction per se, which is an expected adaptive response to oxidative stress, but in demonstrating the functional insufficiency of endogenous Nrf2 signaling and the protective effect of Nrf2 augmentation in CuONP-exacerbated asthma.

Nrf2 functions as a central transcriptional regulator orchestrating cellular antioxidant defenses against oxidative stress and has been implicated in redox signaling, proteostasis, and the modulation of inflammation [49]. Within the inflammatory milieu, Nrf2 is abundantly expressed in monocytes and granulocytes, where it modulates immune responses by regulating antioxidant gene expression and limiting ROS-mediated tissue damage [25]. Elevated local and systemic levels of ROS can disrupt redox signaling and perpetuate inflammation; however, Nrf2 activation interrupts this cycle by restoring redox homeostasis and attenuating downstream inflammatory cascades [50,51]. In our study, AAV-mediated overexpression of Nrf2 markedly alleviated airway hyperresponsiveness, eosinophilic infiltration, mucus hypersecretion, and oxidative stress in CuONP-exposed asthmatic mice. This protective effect is consistent with previous findings that Nrf2 activation suppresses the production of pro-inflammatory cytokines and limits tissue injury under oxidative stress conditions [52,53]. Additionally, in vitro knockdown of Nrf2 in NCI–H292 cells led to enhanced ROS accumulation and upregulation of pro-inflammatory cytokines following CuONP exposure, reinforcing the critical role of Nrf2 in cellular resilience to nanoparticle-induced toxicity. That is, exogenous augmentation of Nrf2 activity may provide a therapeutic advantage by restoring antioxidant defenses and limiting inflammation in nanoparticle-aggravated airway diseases.

Taken together, our findings provide compelling evidence that CuONP exposure exacerbates asthma pathology, characterized by eosinophilic inflammation and mucus hypersecretion, not only by inducing oxidative stress but also by insufficient activation of Nrf2-mediated antioxidant responses. While direct clinical data linking CuONP exposure to these specific histological features in humans remain limited, the pathological profile observed in our study mirrors that of severe human asthma, where oxidative stress and defective antioxidant defenses are hallmarks of disease progression [30]. In this context, the present study is best interpreted as a mechanistic model of disease exacerbation in a susceptible airway background, rather than as a direct simulation of human occupational exposure. Our results suggest that CuONP exposure increased oxidative burden alongside Th2-associated inflammatory endpoints, and AAV-mediated enhancement of Nrf2 signaling attenuated airway inflammation, mucus hypersecretion, and redox imbalance. By demonstrating that exogenous enhancement of Nrf2 activity significantly mitigates these pathological features, this study highlights the pivotal role of the Nrf2 pathway as a potential therapeutic target in nanoparticle-exacerbated asthma. Nevertheless, some limitations should be noted. Although the OVA-induced model effectively reproduces key features of allergic inflammation, potential variability across sensitization protocols and the inherent translational gap between murine models and human asthma should be considered when interpreting the findings. Complementary models such as house dust mite–based or chronic allergen exposure protocols may further improve translational relevance. Another limitation of the study design is the absence of a concurrent CuONP-only group within the OVA-based asthma exacerbation experiments, although CuONP-only pulmonary toxicity was evaluated separately. This limits formal discrimination between additive effects and interaction-driven exacerbation in the allergic airway context. Most in vivo experiments in the present study were also conducted using a single CuONP dose, which limits formal dose–response interpretation. In addition, direct quantitative translation of the intranasal CuONP dose used in this study to occupational airborne exposure limits (e.g., mg/m^3^) is limited, because the experimental design reflects bolus delivery rather than chronic inhalation exposure and does not account for ventilation-dependent deposition or pulmonary clearance kinetics. Although CuONPs induced dose-dependent cytotoxicity in NCI–H292 cells, the specific cell death modalities across exposure concentrations were not determined in the present study. Future studies incorporating multi-dose in vivo designs and dedicated cell death assays will be needed to define concentration-dependent toxicodynamic profiles more precisely. In mechanistic terms, this study focused primarily on Nrf2, and other redox-sensitive signaling pathways, such as NF-κB or AP-1, that may interact with or compensate for Nrf2 were not explored. Moreover, the present study did not dissect upstream redox sensors, copper-specific signaling networks, or potential non-canonical pathways regulating Nrf2 responses under CuONP exposure. Elucidation of these mechanisms will be important to refine the molecular framework of CuONP-induced asthma exacerbation and to identify more selective therapeutic targets. Future studies directly comparing CuONPs with bulk CuO and defining upstream Nrf2-related regulatory mechanisms will be important for clarifying nanoparticle-specific pathways underlying copper oxide–induced airway toxicity. Finally, the long-term effects of CuONP exposure and sustained Nrf2 modulation remain to be established. Further studies are warranted to delineate the broader landscape of oxidative stress-related signaling and to evaluate the therapeutic efficacy of pharmacological Nrf2 activators in chronic exposure models. Such efforts may facilitate the development of more effective strategies for preventing or treating asthma exacerbations triggered by environmental nanoparticles.

In conclusion, this study elucidates a critical mechanistic link between CuONP-driven oxidative stress and asthma exacerbation, mediated by Nrf2-dependent signaling. Although CuONP-induced oxidative stress and Nrf2 activation are broadly consistent with prior literature, the present study extends this framework by demonstrating that endogenous Nrf2 responses are insufficient in CuONP-aggravated asthma and that Nrf2 augmentation mitigates the resulting pathology. Accordingly, strategies aimed at reinforcing endogenous antioxidant defenses, particularly via Nrf2 signaling, may offer a rational approach to limiting nanoparticle-induced exacerbation of airway inflammation and oxidative injury within the respiratory tract.

## Funding

This work was supported by the 10.13039/501100003725National Research Foundation of Korea (NRF) grant funded by the Korea Government (MSIT) (RS-2025-00559511, RS-2023-00219517 and RS-2025-25396564).

## CRediT authorship contribution statement

**Woong-Il Kim:** Formal analysis, Investigation, Methodology, Writing – original draft. **Sin-Hyang Park:** Formal analysis. **Ba-Reun Jin:** Formal analysis. **So-Won Pak:** Formal analysis. **Junhyeong Lee:** Formal analysis. **Min-Jung Park:** Conceptualization. **Changjong Moon:** Conceptualization. **In-Sik Shin:** Conceptualization, Project administration, Supervision. **Jong-Choon Kim:** Conceptualization, Project administration, Supervision, Writing – review & editing.

## Declaration of competing interest

The authors declare that they have no known competing financial interests or personal relationships that could have appeared to influence the work reported in this paper.

## Data Availability

Data will be made available on request.
