# Supplementary material for: Nrf2 pathway mediates copper oxide nanoparticle-induced exacerbation of allergic asthma
Source: Redox Biol. 2026 Apr 20;93:104180. doi: 10.1016/j.redox.2026.104180 (PMC13122667; doi:10.1016/j.redox.2026.104180)
Supplement: Multimedia component 1 [file mmc1.docx]

Supplementary Materials for

**Nrf2 signaling modulates the exacerbation of allergic asthma by copper oxide nanoparticles**

Woong-Il Kim^a^, Sin-Hyang Park^b^, Ba-Reun Jin^b^, So-Won Pak^c^, Junhyeong Lee^b^, Min-Jung Park^b^, Changjong Moon^b^, In-Sik Shin^b, *^, Jong-Choon Kim^b, **^

^a^ *Research Institute of Veterinary Medicine, Chonnam National University, Gwangju 61186, Republic of Korea*

^b^ *College of Veterinary Medicine and BK21 FOUR Program, Chonnam National University, Gwangju 61186, Republic of Korea*

^c^ *Center for Convergence Toxicology Research, Korea Institute of Toxicology, Daejeon 34114, Republic of Korea*

^*, **^ Corresponding author

*E-mail address*: [dvmmk79@gmail.com](mailto:dvmmk79@gmail.com) (I.-S Shin), [toxkim@jnu.ac.kr](mailto:toxkim@jnu.ac.kr) (J.-C Kim)

Additional file: Figures S1–3


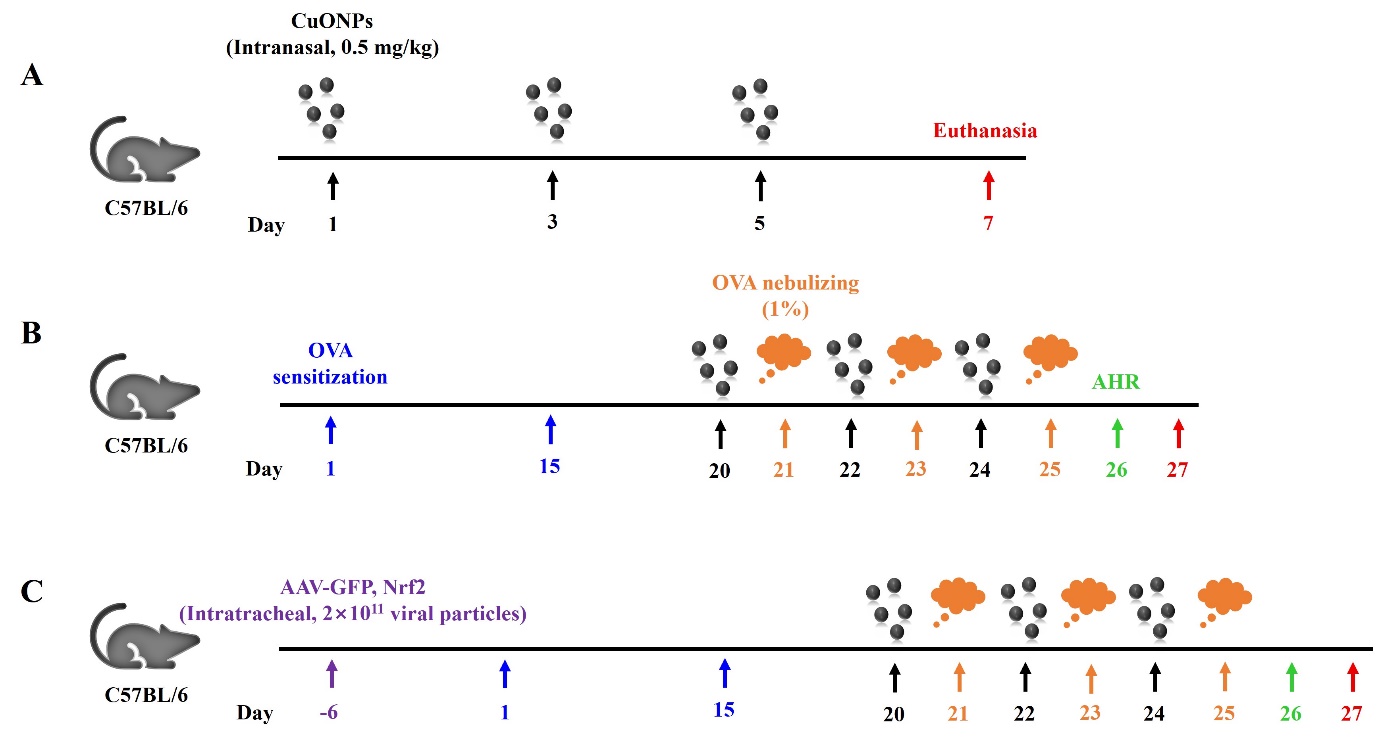


**Fig. S1.** Schematic of the experimental design. (A) Acute CuONPs respiratory toxicity protocol. (B) OVA-induced asthma with CuONP exposure. (C) Nrf2-overexpressing mice subjected to the OVA + CuONPs protocol.


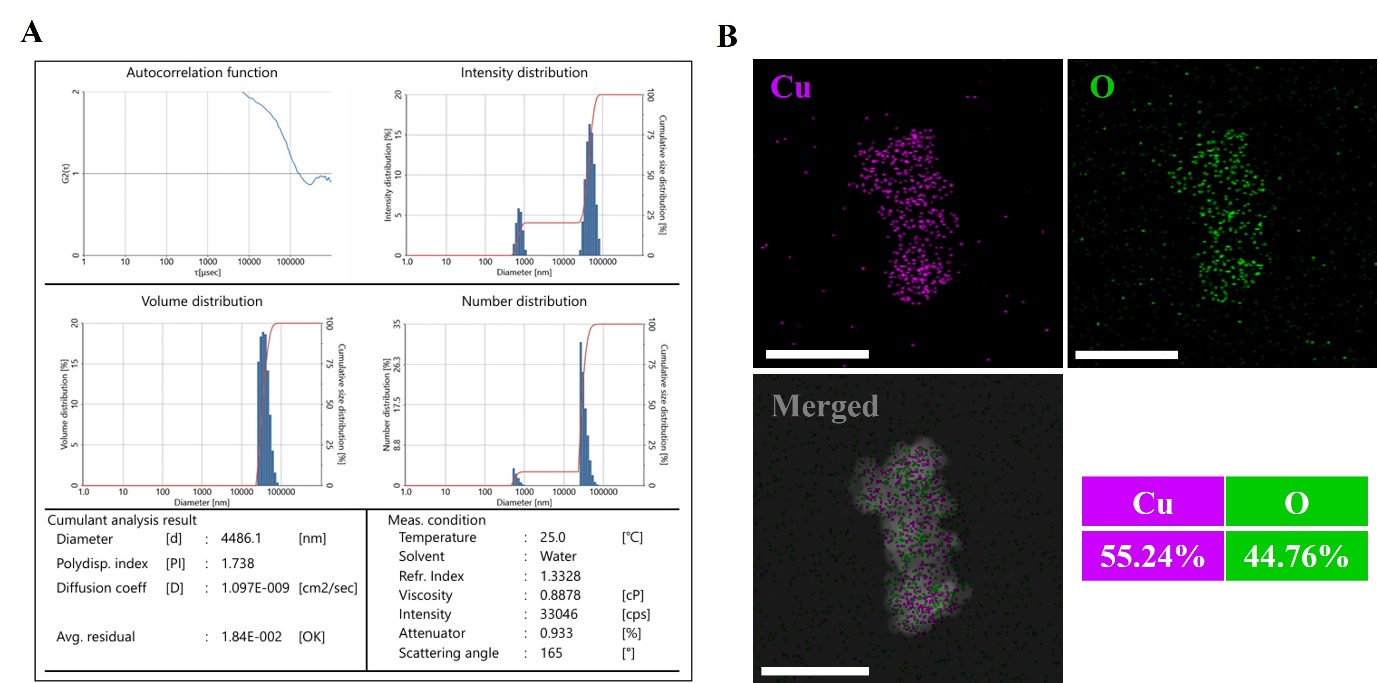


**Fig. S2.** Hydrodynamic size distribution and elemental composition of CuONPs. (A) Hydrodynamic size distribution of CuONPs measured by dynamic light scattering in water at 25 °C (Cumulants mean = 4486.1 nm; PDI = 1.738). (B) Elemental composition of CuONPs assessed by energy-dispersive X-ray spectroscopy (Cu: 55.24 at. %, O: 44.76 at. %; Scale bar = 250 nm).


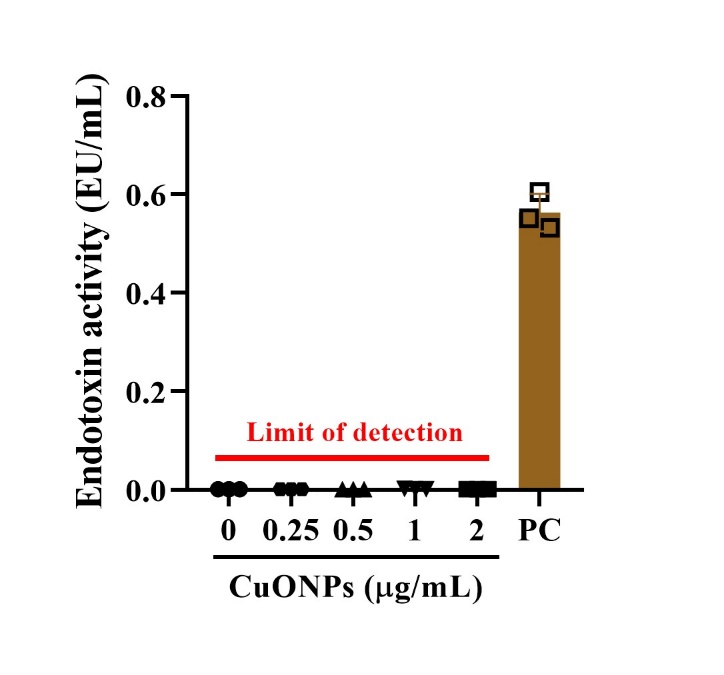


**Fig. S3.** Endotoxin activity (EU/mL) was assessed in CuONP suspensions at 0.25–2 μg/mL using the recombinant Factor C (rFC) assay. Endotoxin (0.5 EU/mL) was used as a positive control (PC). All CuONP samples showed endotoxin activity below the limit of detection (LOD; 0.005 EU/mL). Data are presented as mean ± SD (n = 3).
